# Biomechanical comparison of different prosthetic reconstructions in total en bloc spondylectomy: a finite element study

**DOI:** 10.1186/s12891-022-05919-0

**Published:** 2022-11-04

**Authors:** Hanpeng Xu, Xiaodong Wang, Ye Han, Yuanyuan Jiang, Jianzhong Wang, Xiong Zhang, Jun Miao

**Affiliations:** 1 Tianjin Hospital, Tianjin University, Tianjin, China; 2 Department of Orthopaedics, Affiliated Hospital of Hebei University, Baoding, China; 3 Department of Anesthesiology, Affiliated Hospital of Hebei University, Baoding, China; 4 Tianjin Hospital, Tianjin University, Jiefangnanlu 406, Hexi District, Tianjin, 300210 China

**Keywords:** TES, 3D-printed prosthesis, Titanium mesh cage, Finite element analysis

## Abstract

**Objective:**

To analyse and compare the biomechanical differences between 3D-printed prostheses, titanium mesh cages and poorly matched titanium mesh cages in total en bloc spondylectomy (TES).

**Methods:**

The finite element model of T10-L2 for healthy adults was modified to make three models after T12 total spondylectomy. These models were a 3D-printed prosthesis, titanium mesh cage and prosthesis-endplate mismatched titanium mesh cage for reconstruction. The range of motion (ROM), stress distribution of the endplate and internal fixation system of three models in flexion and extension, lateral bending and axial rotation were simulated and analysed by ABAQUS.

**Result:**

In flexion, due to the support of the anterior prosthesis, the fixation system showed the maximum fixation strength. The fixation strength of the 3D-printed prosthesis model was 26.73 N·m /°, that of the TMC support model was 27.20 N·m /°, and that of the poorly matched TMC model was 24.16 N·m /°. In flexion, the L1 upper endplate stress of the poorly matched TMC model was 35.5% and 49.6% higher than that of the TMC and 3D-printed prosthesis, respectively. It was 17% and 28.1% higher in extension, 39.3% and 42.5% higher in lateral bending, and 82.9% and 91.2% higher in axial rotation, respectively. The lower endplate of T11 showed a similar trend, but the magnitude of the stress change was reduced. In the stress analysis of the 3D-printed prosthesis and TMC, it was found that the maximum stress was in flexion and axial rotation, followed by left and right bending, and the least stress was in extension. However, the mismatched TMC withstood the maximum von Mises stress of 418.7 MPa (almost twice as much as the buckling state) in rotation, 3 times and 5.83 times in extension, and 1.29 and 2.85 times in lateral bending, respectively.

**Conclusion:**

Different prostheses with good endplate matching after total spondylectomy can obtain effective postoperative stable support, and the reduction in contact area caused by mismatch will affect the biomechanical properties and increase the probability of internal fixation failure.

## Introduction

Total en bloc spondylectomy (TES) is considered an effective method for the treatment of primary spinal tumours and selective metastatic spinal tumours, but 360° stabilization reconstruction is required after TES to restore spinal function. Biomechanical studies show that posterior multilevel pedicle screw fixation and anterior vertebrae replacement (VBR) support are required to restore stability after TES [1–3]. There are various options for anterior VBR [4]. At present, the most common clinical application is still a titanium mesh cage (TMC) combined with autologous and allogeneic bone materials. There are various choices of different diameters and lengths for adapting to the cervicothoracic and lumbar spine, and they can be tailored according to patient needs. However, trimmed TMCs cannot match the shape of the endplate and the sagittal alignment of the spine (lordosis/kyphosis), resulting in reduced contact area, stress concentration, TMC subsidence and even instrument failure [5].

The application of 3D-printed prostheses has received widespread attention in recent years. It can be perfectly matched to the adjacent endplate by computer scanning, and the resulting porous prosthesis is suitable for osteocyte ingrowth, precluding the need for grafted bone application. At present, there have been many reports [6–8], and good application results have been obtained. However, there is no relevant report on the biomechanical differences between 3D-printed prostheses and TMCs, especially the biomechanical difference caused by the poor matching of the TMC with the endplate.

Therefore, the aims of our study were 1) to evaluate whether 3D-printed prostheses could result in improved biomechanical properties compared with TMCs after TES and 2) to evaluate the biomechanical properties correlated with prosthesis-endplate mismatch differences.

## Materials and methods

### Intact thoracolumbar finite element model

We selected a healthy 32-year-old male volunteer (172 cm, 72 kg) to create a finite element model. The volunteer had no spinal diseases, spinal trauma or surgery and had no obvious degenerative disease after X-ray examination of the thoracic and lumbar spine.

CT data scans of the thoracolumbar segment were performed on volunteers, and a 64-slice spiral computed tomography scanner (Siemens, Erlangen, Germany) was used to scan with a tube voltage of 120 kV, a tube current of 200 mA, and a slice interval of 1 mm. Image data of 5 vertebral bodies and 4 intervertebral discs were obtained between T10 and L2. Mimics 20.0 (Materials Company of Leuven, Belgium) was applied to draw the precise vertebral body and perform 3D image calculations to create five 3-dimensional (3D) vertebral body surface models from T10 to L2 and generate STL format files.

The facet joint, annulus fibrosus, and nucleus pulposus were constructed using 3-Matic 12.0 software (Materialise Inc.) [9–11]. Then, they were imported into Geomagic Studio 2015 (Geomagic NC).Bone, disc and ligament structures were meshed using Hypermesh2017 (Altair Engineering, Troy, MI, USA). Abaqus2020 (Simulia, Johnston, RI, USA) was used to perform model assembly and then add material properties, loading loads and finite element analysis.

**Fig. 1:**
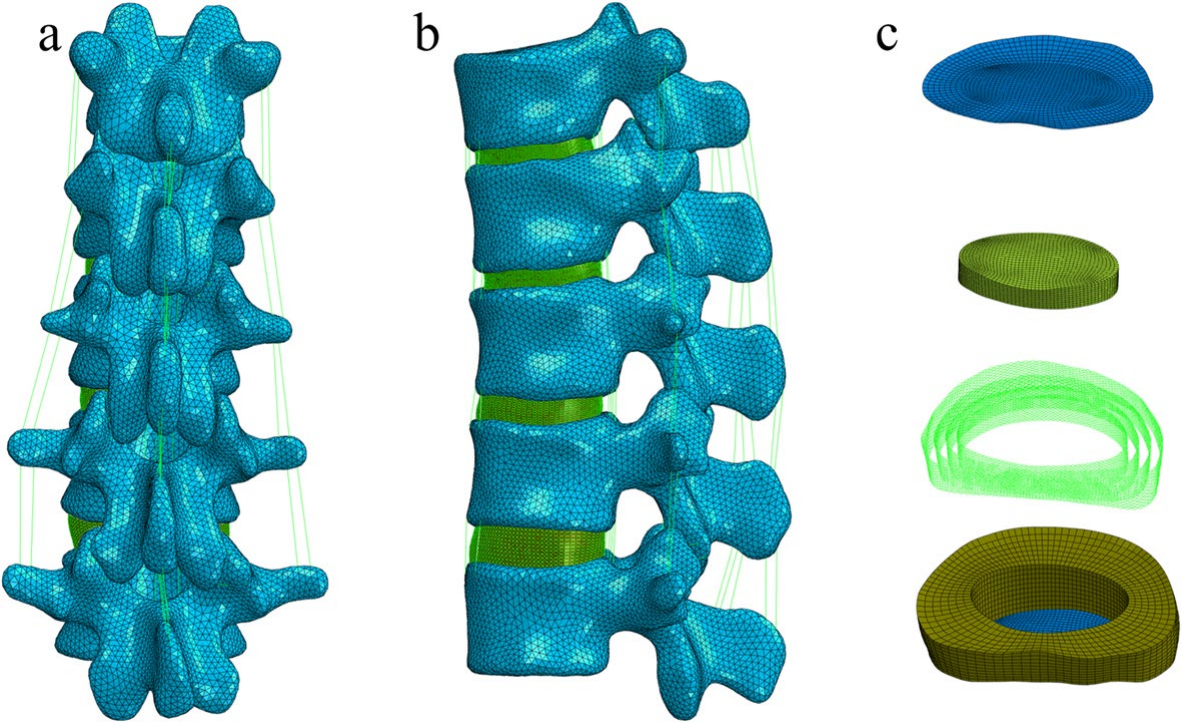
**a**, **b**. Posterior and lateral view of the thoracolumbar finite element model (including five vertebrae, four intervertebral discs, anterior and posterior longitudinal ligaments, interspinous process, superior spinous process ligament and ligamentum flavum, etc.). **c** Intervertebral disc (including nucleus pulposus, annulus fibrosus matrix and reinforced annulus fibrosus)

The intact T10-L2 thoracolumbar finite element model is shown in Fig. 1 (a, b). The cortical shell, facet joints, and cartilage endplates were modelled with shell elements with thicknesses of 1 mm, 0.2 mm, and 0.5 mm, respectively [9, 10, 12]. The disc was divided into the nucleus pulposus and annulus fibrosus. The nucleus pulposus accounted for 30% to 40% of the intervertebral volume, as shown in Fig. 1(c). The annulus fibrosus consisted of an annulus fibrosus matrix and reinforcing collagen fibres, which were generated in 3–5 layers at a 30-degree angle from the horizontal surface [1, 3]. The truss element was used for the ligaments and fibrosus. Each segment simulated 7 ligaments: the anterior longitudinal ligament (ALL), the posterior longitudinal ligament (PLL), the ligamentum flavum (LF), the capsular ligament (CL), the intertransverse ligament (ITL), the interspinous ligament (ISL) and the supraspinous ligament (SSL) [9, 13].

The maximum von Mises stress of the model with element size of 1.5 mm, 2.0 mm and 2.5 mm is calculated and compared with the model with element size of 1.0 mm. When the difference is less than 5%, the element is considered to be convergent. In terms of the load and calculation accuracy, the element size of 1.5 mm is selected. In this case, the percentage error is 3.51%. The complete T10-L2 finite element model had a total of 410,764 elements.

### Validation of the finite element model

Spinal movements in sagittal, coronal, and transverse planes were defined as flexion and extension, lateral bending, and rotation, respectively. The lower surface of the L2 vertebral body was fixed, and a pure moment of 7.5 Nm was applied to the upper surface of T12. The range of motion (ROM) was measured and compared with previous reports [14–16].

### Finite element postoperative model

SolidWorks software (Dassault Systems, Paris, France) was used to draw the finite element model of the internal fixator, pedicle screws (6.0 × 40 mm), rods (5.5 mm), titanium mesh cages (Medtronic, USA) and artificial 3D-printed prosthesis (AK Medical, Beijing, China), which were meshed using Hypermesh2017. The dimension of the 3D-printed prosthesis and TMC is 20 mm. The 3D-printed prosthesis is a porous structure made of titanium alloy (Ti6Al4V). The effective Young’s modulus was used to characterize the material property of the porous structure for simplification [17, 18]. The material properties used in the finite element model (Table 1) are based on previous reports [19, 20].

**Table 1:** Material properties of the thoracolumbar spine model and internal fixation

Structure	Young’s modulus (MPa)	Poisson’s ratio	Cross-section area (mm^2^)
Vertebrae			
Cancellous bone	100	0.2	
Cortical bone	12,000	0.3	
Posterior elements	3500	0.25	
Disc			
Annulus	4.2	0.45	
Nucleus	0.2	0.49	
Facet	11	0.2	
Ligaments			
ALL	7		63.7
PLL	7		20
LF	3		40
ITL	7		1.8
CL	4		30
ISL	6		40
SSL	6.6		30
Pedicle screw、 rod fixation and Mesh cage	110,000	0.3	
3D printed prosthesis	675	0.3	
Bone graft	100	0.2	

**Fig. 2:**
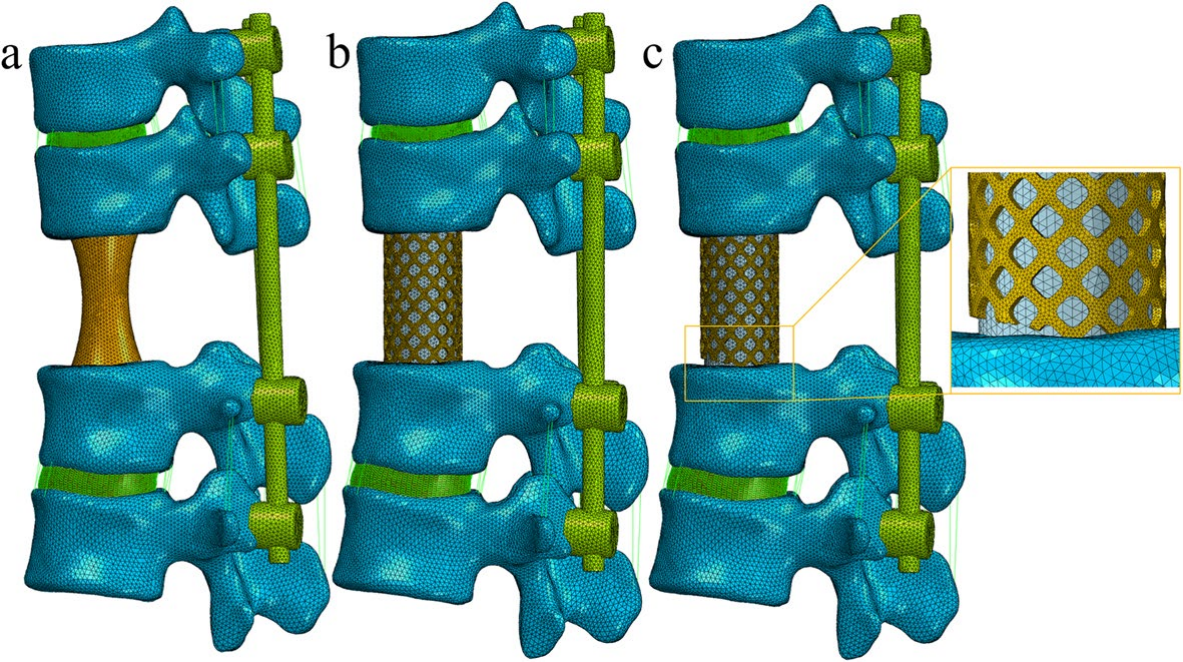
**a**. Model of 3D-printed prosthesis reconstruction after T12 TES; (**b**) Model of titanium mesh cage reconstruction after T12 TES; (**c**) Model and local magnification of mismatched titanium mesh cage reconstruction after T12 TES

Figure 2 shows three finite element models of T12 TES. The intact vertebrae of T12 and the T11-12 and T12-L1 discs and the corresponding anterior and posterior longitudinal ligaments were removed. The models were fixed with posterior fixation (two-level pedicle screw fixation in the upper and lower VBR; T10/11 and L1/2) and different VBRs. In model A, the VBR is a 3D-printed prosthesis; in model B, the VBR is a TMC that is well matched to the endplate; and in model C, the front 1/3 diameter of the TMC contact with the endplate of L1 is removed to simulate a situation where the TMC is mismatched to the endplate. The TMC was filled with bone graft, and the material properties used was listed in Table 1. Two 6.0 × 40 mm pedicle screws were inserted into T10 and T11, and two 6.5 × 45 mm pedicle screws were inserted into L1 and L2 for posterior fixation. Rigid connections were formed among cage and bone, bone and screws, screws and rods by using the ‘Tie Contact’ feature in ABAQUS. T12 TES finite element models with three different VBRs were successfully fabricated (Fig. 2).

### Finite element simulation analysis

Abaqus 2020 (Abaqus Inc., USA) was used to evaluate boundary and load conditions as well as the simulation of spinal motion. We assumed that L2 was fixed and set its substructure as a boundary with no displacement or rotation in all directions. Spinal motion in the sagittal, coronal, and transverse planes was defined as flexion, extension, and rotation, respectively. An axial load of 200 N and an additional torque load of 7.5 N·m were applied to simulate flexion, extension, and rotation of the spine, according to the human body's bearing capacity and previously published literature [15]. Loads were applied to the upper surface of the T10 vertebra.

## Results

### Model validation

**Fig. 3:**
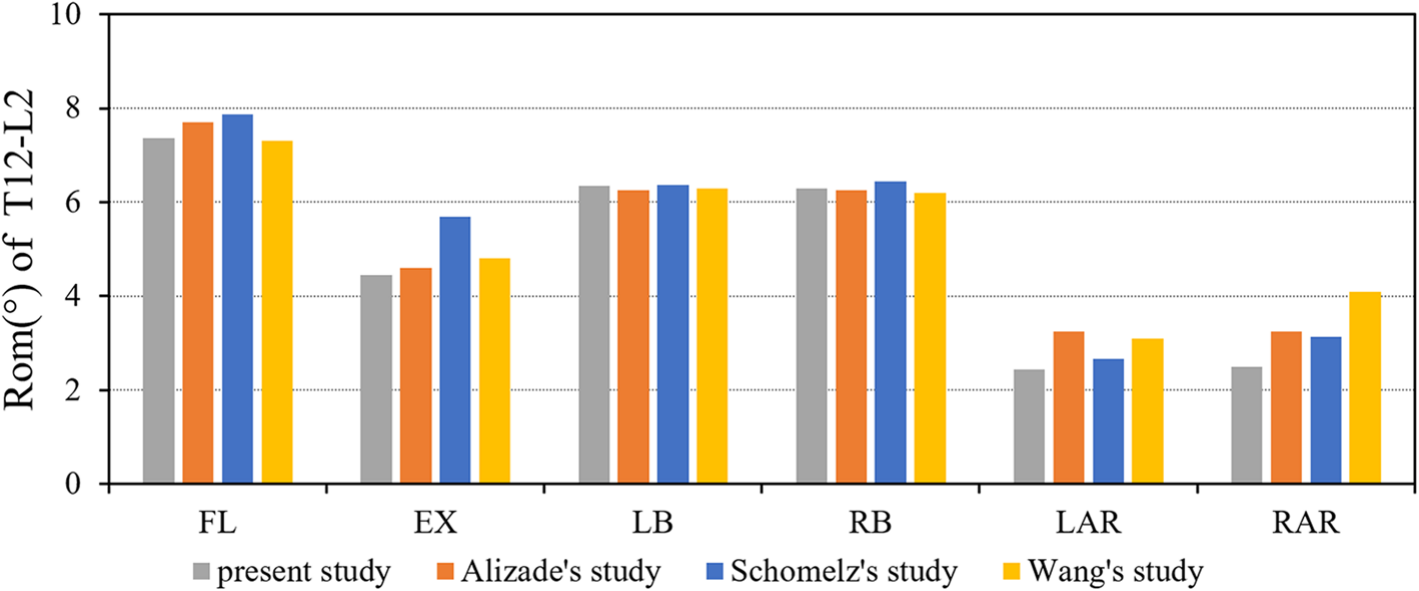
ROM of the T12-L2 in intact finite element model made in this study is compared with previously reported data

ROM measurements of the flexion, extension, lateral bending, and axial rotation of the intact thoracolumbar model (T12-L2) were compared with results from previous biomechanical studies and finite element models[14–16] (Fig. 3). The predicted range of motion of the lumbar segment is basically consistent with the previous experimental data.

### Fixation strength of fixed segments under different prosthesis supports

**Fig. 4:**
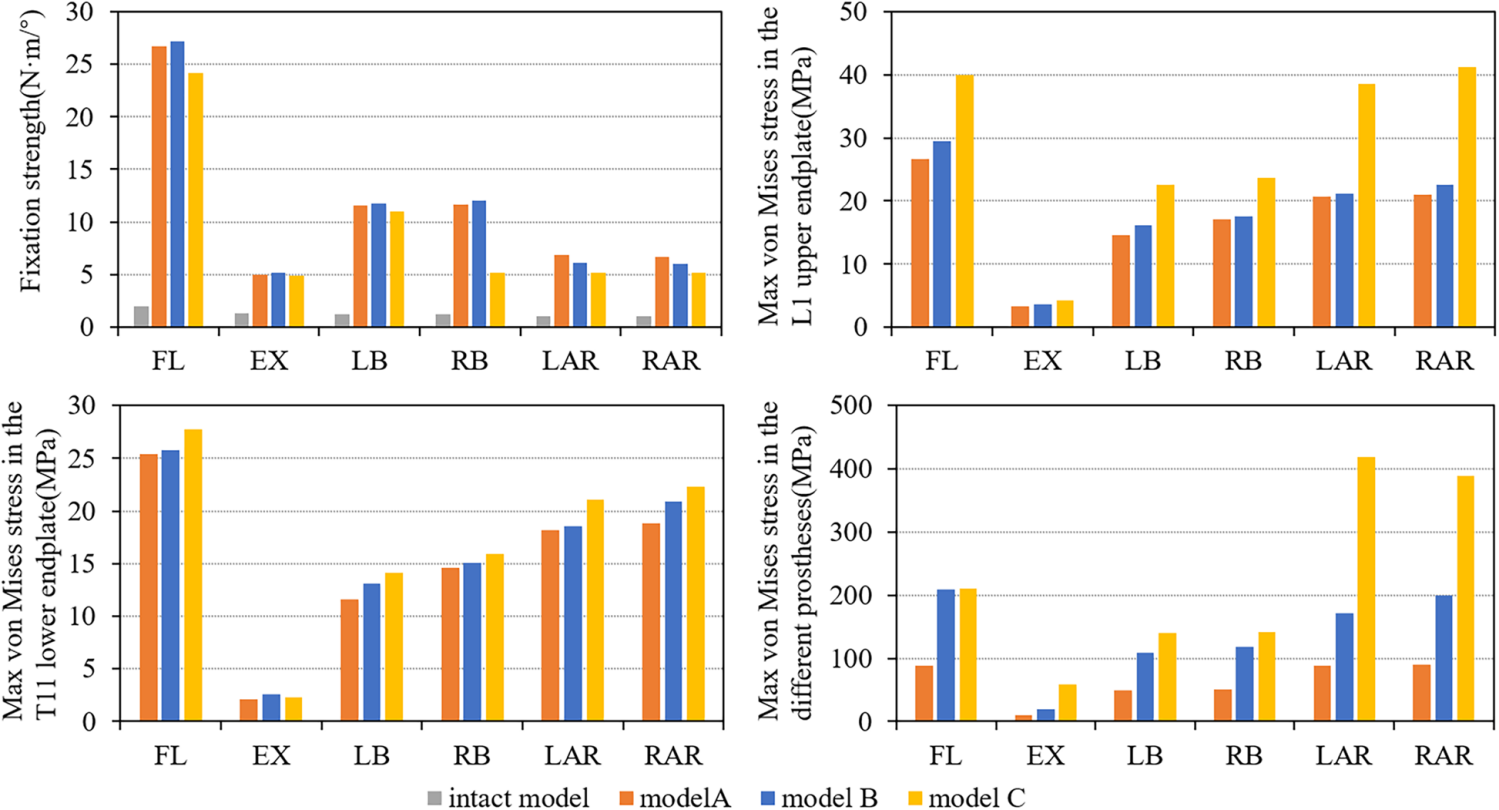
Fixation strength, maximum von Mises stress of L1 upper endplate, T11 lower endplate and different prostheses in flexion (FL), extension (EX), left lateral bending (LB), right lateral bending (RB), left axial rotation (LAR), right axial rotation (RAR)

All three prosthetic supports showed much higher fixation strengths than the full model under the tested loading conditions. In flexion, due to the support of the anterior prosthesis, the fixation system showed the maximum fixation strength. The fixation strength of the 3D-printed prosthesis model was 26.73 N·m /°, that of the TMC support model was 27.20 N·m /°, and that of the poorly matched TMC model was 24.16 N·m /°. The three models had similar fixation strengths in flexion, extension, and lateral bending, and there was no difference. In rotation, the 3D-printed prosthesis model has the highest fixed strength, and the fixed strengths of the left and right rotations were 6.83 N·m /° and 6.68 N·m /°, respectively. These values were increased by 11.29% and 30.78%, respectively, compared with 6.16 N·m /° and 5.98 N·m /° for the TMC model and 5.18 N·m /° and 5.15 N·m /° for the poorly matched TMC support model (Fig. 4).

### Von Mises stress of the adjacent endplate of the prosthesis under different prosthesis supports

**Fig. 5:**
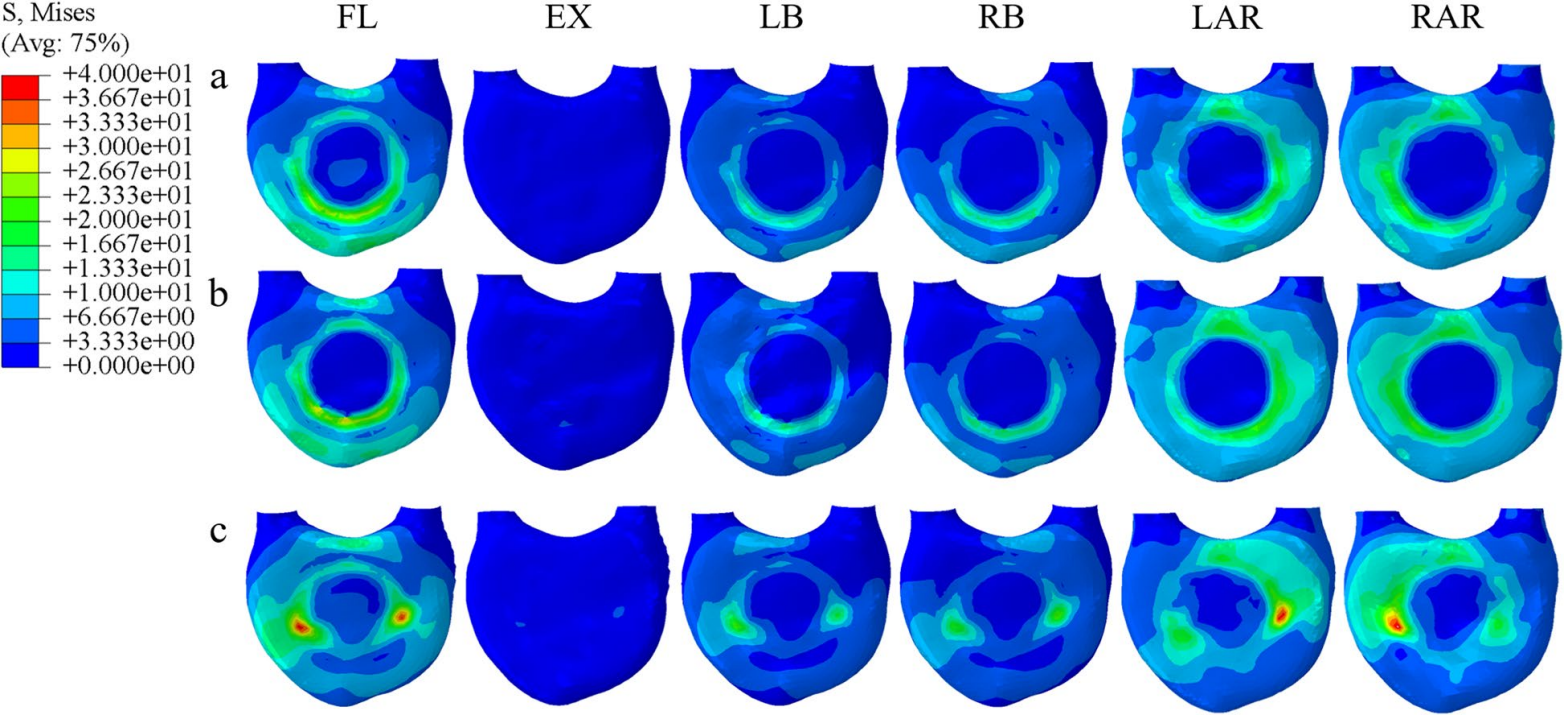
Von Mises stress of the upper endplate of L1 supported by three kinds of prostheses: (**a**) 3D-printed prosthesis; (**b**) titanium mesh cage; (**c**) mismatched titanium mesh cage (FL: flexion, EX: extension, LB: left lateral bending, RB: right lateral bending, LAR: left axial rotation, RAR: right axial rotation)

**Fig. 6:**
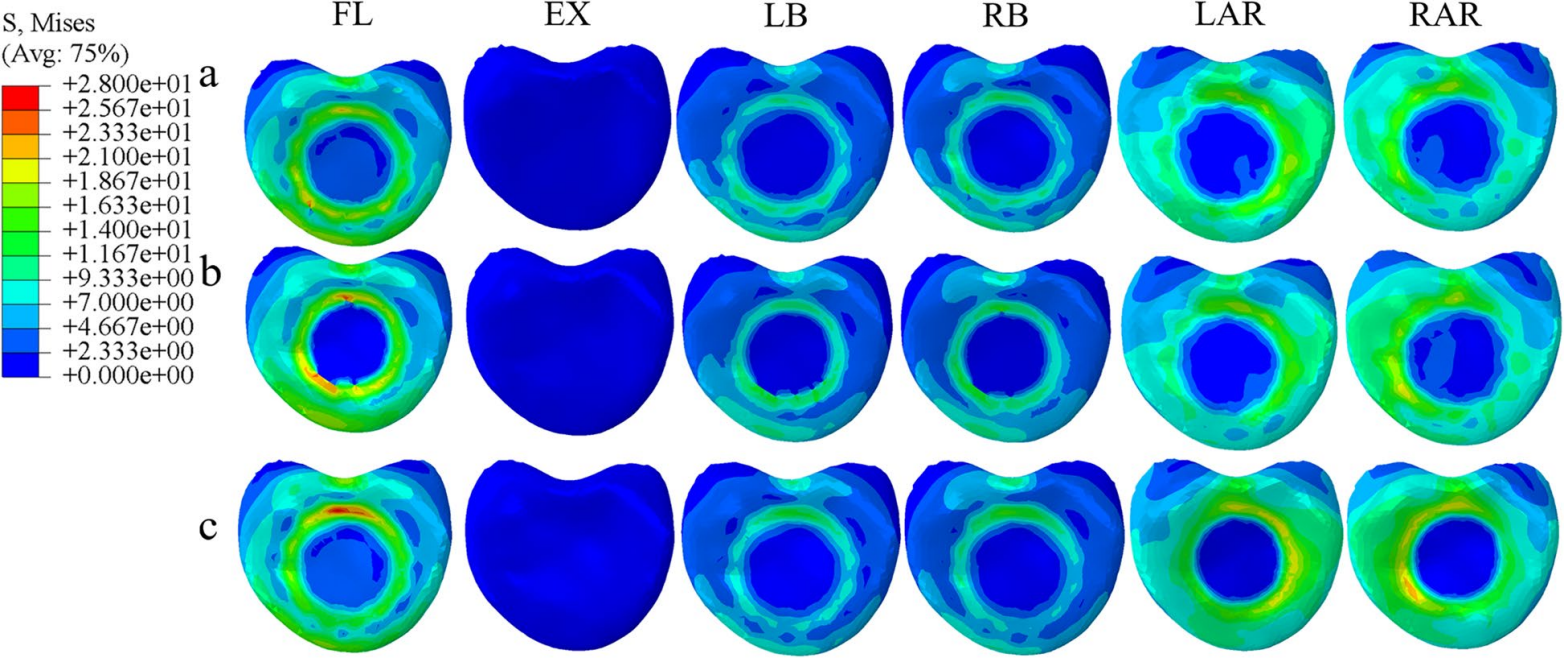
Von Mises stress of the lower endplate of T11 supported by three kinds of prostheses: (**a**) 3D-printed prosthesis; (**b**) titanium mesh cage; (**c**) mismatched titanium mesh cage (FL: flexion, EX: extension, LB: left lateral bending, RB: right lateral bending, LAR: left axial rotation, RAR: right axial rotation)

The von Mises stress of the three fixed models in the adjacent endplate of the prosthesis showed that the stress of the L1 upper endplate was higher than that of the T11 lower endplate. In flexion, the L1 upper endplate stress of the poorly matched TMC model was 35.5% and 49.6% higher than that of the TMC and 3D-printed prosthesis, respectively. It was 17% and 28.1% higher in extension, 39.3% and 42.5% higher in lateral bending, and 82.9% and 91.2% higher in axial rotation, respectively, and the stress concentration of the mismatched TMC was observed in all motions (Fig. 5). The lower endplate of T11 showed a similar trend, but the magnitude of the stress change was reduced (Fig. 6).

### Von Mises stress of different prostheses

**Fig. 7:**
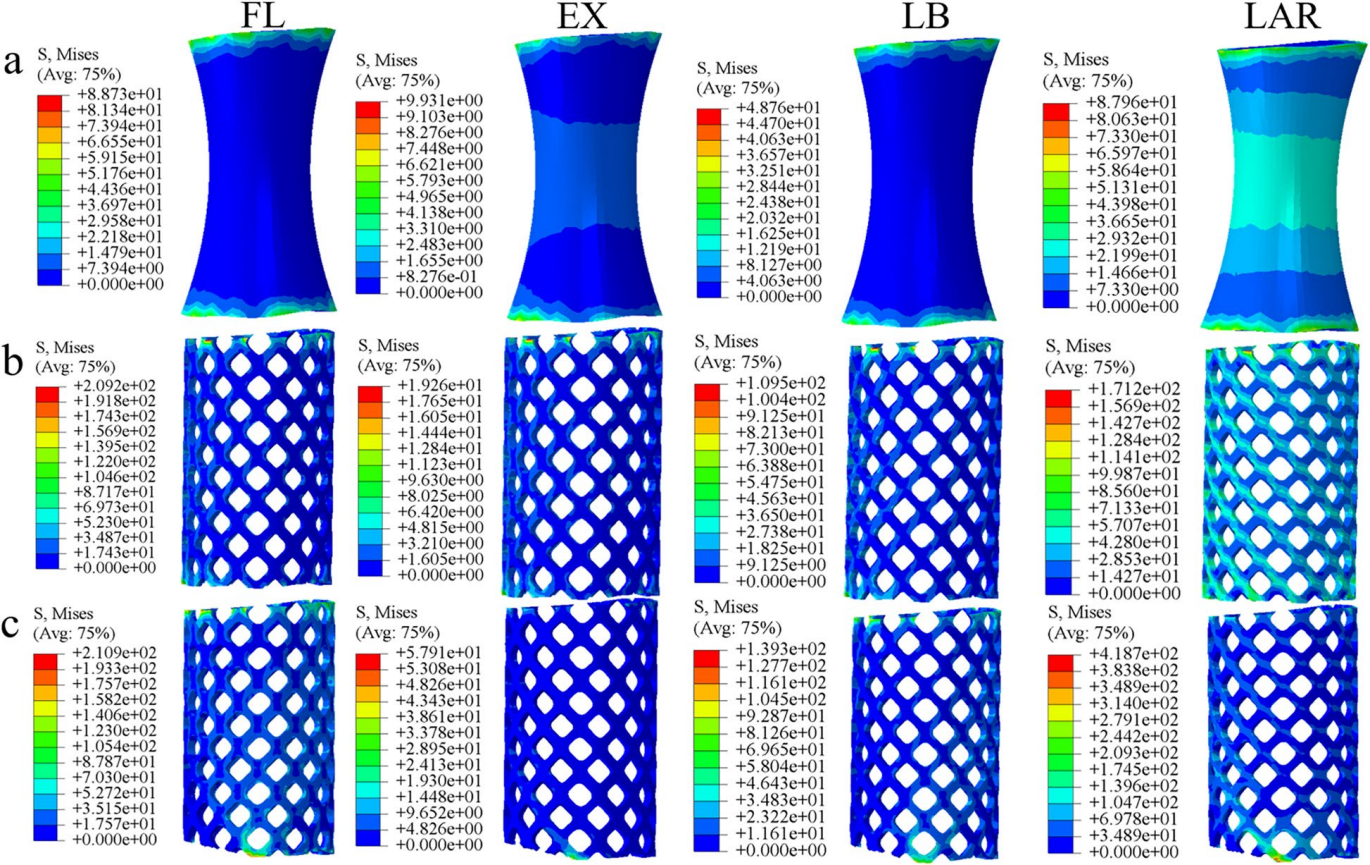
Von Mises stress of the three prostheses in different motion states: (**a**) 3D-printed prosthesis; (**b**) titanium mesh cage; (**c**) mismatched titanium mesh cage(FL: flexion, EX: extension, LB: left lateral bending, LAR: left axial rotation)

Under different anterior prosthesis supports, the maximum stress on the prosthesis also showed differences. In the stress analysis of the 3D-printed prosthesis and TMC, it was found that the maximum stress was in flexion and axial rotation, followed by left and right bending, and the least stress was in extension. However, the mismatched TMC withstood the maximum von Mises stress of 418.7 MPa (almost twice as much as the buckling state) in rotation, 3 times and 5.83 times in extension, and 1.29 and 2.85 times in lateral bending, respectively. In flexion, the stress of the mismatched TMC was similar to that of the TMC, which was approximately 2.74 times that of the 3D printing prosthesis. Generally, the smaller the contact area between the anterior prosthesis and the endplate, the greater the force on the prosthesis, and there was a stress concentration in the mismatched TMC prosthesis, while the well-matched TMC and 3D-printed prosthesis had uniform stress distributions. The position of the maximum force also changed in different motion states, but mainly at the lower end of the prosthesis. (Fig. 7).

## Discussion

In the selective treatment of spinal tumours, total spondylectomy can improve the quality of life and even prolong the survival time of patients [21]. The literature shows that the thoracic vertebrae, especially the lower thoracic vertebrae, are the most common sites of metastatic tumours [22].

Considering that total spondylectomy is indicated for patients with a longer life expectancy [23], firm spinal reconstruction is essential for the patient's long-term quality of life. The literature report shows that the mismatch between the implanted TMC and the endplate after thoracolumbar spine resection leads to a reduction in the contact area, which is the main factor for prosthesis subsidence, internal fixation failure and even fracture [24].

An artificial vertebral body manufactured by 3D printing can achieve good prosthesis-endplate matching and increase the contact area, which is considered to yield biomechanical properties. Moreover, the porous structure reduces the elastic modulus of the metal and reduces the stress concentration at the prosthesis-bone interface, which can also contribute to obtaining better biomechanical properties. However, there is no relevant biomechanical experimental evidence to support this at present. In this experiment, finite element analysis was used to simulate the stability and mechanical analysis of three kinds of front-supported prostheses: 3D-printed prostheses, well-matched TMCs and mismatched TMCs. The results showed that the anterior support of the three prostheses achieved good immediate stability. The application of 3D-printed prostheses increased the contact area with the adjacent endplate, reduces the pressure of the adjacent endplate, and enhances the stability of the fixation system, especially the rotational stability. When the mismatched TMC was used, the stress of the adjacent endplate and the force of the TMC increased, which became an important reason for the sinking of the TMC. On the other hand, when the well-matched TMC was well supported, the stress of the adjacent endplate was between that of the above two kinds of prostheses, which also indicated good stability.

### Effects of different prostheses on spinal segmental fixation strength after total spondylectomy

In this paper, we used the strength of the fixed structure to represent the degree of stability after fixation. The results showed that the three fixation models after total spondylectomy had good initial stability, which was higher than that of the complete thoracolumbar model. This is consistent with the results of previous biomechanical experiments [1–3]. Moreover, our research showed that the maximum fixation strength was obtained by using a TMC as the front support except for rotation, while the fixation strength of the TMC with poor matching with the endplate was the worst. The segmental fixation strength of the three kinds of prostheses showed little difference, and there was no difference. In the rotating state, the support stability of the 3D-printed prosthesis was enhanced, and its fixation strength was increased by 12.9% and 39.3% compared with that of the TMC and the mismatched TMC, respectively, indicating that the increase in the contact area with the endplate enhanced the rotational stability of the fixation system. A number of previous biomechanical studies have shown that the firm fixation of surgical segments after total thoracolumbar vertebrae surgery is mainly attributed to the fixation of posterior long segment pedicle screws and anterior prosthetic support [25, 26]. Pflugmacher [27] et al. used different lengths of posterior segment fixation and anterior retractable prostheses and TMCs as support in a study of L1 vertebral resection. The results showed that only the length of the posterior fixed segment affected the overall fixation strength of the spine, and different kinds of anterior support did not lead to biomechanical differences. However, the above experiments ignore the differences in stability caused by different contact modes between the prosthesis and the adjacent endplate. Moreover, the straight thoracolumbar spine was used as the experimental object, ignoring the biomechanical differences caused by different prostheses matching the thoracic and lumbar segments with physiological curvature. However, in cervico-thoracolumbar vertebrae resection, different physiological curvatures often lead to mismatch of anterior implant prostheses after vertebrae resection, which affects the stability of postoperative reonstruction [5, 24]. Although we used a straight thoracolumbar model in our experiment, we simulated the mismatch of the prosthesis-endplate caused by the curvature of the spine and the rough cutting of the TMC. The results showed that different prostheses led to different degrees of matching with the endplate, and the difference in spinal stability caused by different contact areas was substantial, especially in the state of rotation. These results show that anterior prosthesis-endplate matching plays an important role in spinal stability reconstruction.

In the stability analysis, the TMC with good matching obtained the maximum fixed strength in the state of flexion, extension and lateral bending. The main reason for this phenomenon may be that the strength of titanium alloy is much higher, and the elastic modulus of titanium alloy is very high [28, 29]. The 3D-printed prosthesis we used was composed of a porous titanium alloy structure with a diameter of 800 microns, which greatly reduced the elastic modulus of titanium alloy and was closer to the elastic modulus of bone tissue [8, 30]. Therefore, under pressure, the front column is more likely to exhibit small deformation. However, the fixation strength of the mismatched TMC was lower than that of the other two kinds of prostheses due to the uneven force. However, with the increase in the contact area between the front supporting prosthesis and the adjacent endplate, although the elastic modulus decreased, a fixed strength in all directions similar to that of the stronger TMC was obtained, and the rotational stability increased. The decrease in elastic modulus can also improve the stress of the endplate.

### Effects of implanted prostheses on the stress of the adjacent endplate after total spondylectomy

The stress state of the interface between the implant and the adjacent endplate is very important to ensure the safety of the internal fixation. Excessive endplate stress can lead to fracture and prosthesis sinking. The literature shows that the failure rate of the TMC as an anterior support internal fixation after total spondylectomy can be up to 40% [24, 31], mainly due to the stress concentration caused by the cutting of the endplate and the tilt of the TMC [5]. In this experiment, we simulated the endplate stress of the three prostheses after T12 TES. The results showed that compared with 3D-printed prostheses and TMCs, the stress of the L1 cranial endplate of the model with mismatched TMCs increased in all directions, especially in the state of rotation, and the stress almost doubled. This fully explains why a smaller prosthesis-endplate contact area and excessive body weight (BMI index) will lead to an increase in endplate stress and the sinking of the implant prosthesis.

The 3D-printed prosthesis can be perfectly matched with the adjacent endplate by computer scanning, which increases the contact area of the prosthesis-endplate to increases the stability of the fixed system. Moreover, because it has a porous structure that can grow into the bone, a larger contact area can result in a better environment for bone growth. It is considered to be an ideal implant material after vertebrotomy. In this study, it is also shown that when using the 3D-printed prosthesis as the front support material, a better stress distribution can be obtained by increasing the contact area between the prosthesis and the adjacent endplate, especially on the L1 side, while the literature shows that the prosthesis sinks mainly at the tail end of the prosthesis. Therefore, the application of 3D-printed prostheses is considered to be effective in reducing the probability of prosthesis sinking. Hua Zhou [32] reported that a retrospective study of 23 cases of 3D printed prosthesis reconstruction after thoracolumbar resection showed that only 2 cases had substantial subsidence. It also confirms the excellent performance of 3D printing prosthesis applications.

### Effects of different prosthesis implantation methods on the stress of the internal fixation system after total spondylectomy

In the stress analysis of the three kinds of front prostheses, the 3D-printed prosthesis endured the least stress, while the two kinds of TMCs endured larger prosthesis stress, and their peak stress was much higher than that of the 3D-printed prosthesis. In particular, a large amount of stress concentration was observed at the limited contact point of the mismatched TMC (Fig. 7). The maximum stress of 418.7 MPa was obtained under rotation, which was 2.18 times and 4.54 times higher than that of the well-matched TMC and 3D-printed prosthesis, respectively. During extension, the stress of the mismatched TMC was 3 times and 5.83 times higher than that of the other two kinds of TMCs, respectively. This may be caused by the smaller contact area and the backwards shift of the centre of gravity of the prosthesis, which makes the stress more concentrated during extension. Much higher stress peaks were detected for both TMCs in all states, which may help explain the incidence of near endplate fracture and subsidence after TMC support [18]. There may be two main reasons why the peak stress of the 3D-printed prosthesis is much lower. First, the material of the 3D-printed prosthesis has a similar elastic modulus to our human cortical bone, which can effectively reduce the stress shielding effect. The other is the morphological design of the 3D printing prosthesis, which expands the contact surface with the endplate and helps to disperse the stress on the prosthesis. Because the 3D-printed prosthesis has some superior biomechanical properties compared to the two kinds of TMCs, it can be used as a good choice for spinal stability reconstruction.

This study has several limitations. First, in the finite element analysis, only linear elastic materials were used for the vertebral body and the intervertebral disc. However, the main conclusions of this paper were based on the comparative analysis among three models, thereby being less influenced by the aforementioned simplifications. Moreover, the boundary conditions are simplified. Second, the finite element modelling data obtained from individual-image data may have deviations between individual differences for the whole population. Finally, it is difficult to simulate intervertebral disc degeneration and facet joint disease with a finite element model, but many patients often have spinal degeneration. However, we believe that these effects have little impact on the results because spinal degeneration after total spondylectomy has a limited impact on the stability of the surgical structure. In addition, finite element analysis represents the overall trend, and accurate data need to be further combined with biomechanical tests.

## Conclusion

The stress change of the fixation system is obvious when different prostheses are used for anterior support after total spondylectomy. The mismatch between the TMC prosthesis and adjacent endplate increased the probability of implant subsidence and related complications of the internal fixation system. TMCs and 3D-printed prostheses with the same contact area can obtain good biomechanical properties, but 3D-printed prostheses result in a better fixation strength and lower endplate contact stress and prosthesis stress. Therefore, different prostheses with good matching with the endplate after total spondylectomy can obtain effective postoperative stable support, and the reduction in contact area caused by mismatch will affect the biomechanical properties and increase the probability of internal fixation failure.

## Data Availability

The datasets generated and analyzed during the current study are available from the corresponding author on reasonable request.

## Abbreviations

TES: Total en bloc spondylectomy
VBR: Vertebrae replacement
TMC: Titanium mesh cage
3D: 3-Dimensional
ROM: Range of motion
ALL: Anterior longitudinal ligament
PLL: Posterior longitudinal ligament
LF: Ligamentum flavum
CL: Capsular ligament
ITL: Intertransverse ligament
ISL: Interspinous ligament
SSL: Supraspinous ligament
FL: Flexion
EX: Extension
LB: Left lateral bending
RB: Right lateral bending
LAR: Left axial rotation
RAR: Right axial rotation
